# MCL1 binds and negatively regulates the transcriptional function of tumor suppressor p73

**DOI:** 10.1038/s41419-020-03068-7

**Published:** 2020-11-03

**Authors:** Hayley Widden, Aneta Kaczmarczyk, Ashok Subedi, Robert H. Whitaker, William J. Placzek

**Affiliations:** Department of Biochemistry and Molecular Genetics, University of Alabama at Birmingham, Birmingham, AL USA

**Keywords:** Solution-state NMR, Apoptosis, Stress signalling

## Abstract

MCL1, an anti-apoptotic protein that controls chemosensitivity and cell fate through its regulation of intrinsic apoptosis, has been identified as a high-impact target in anti-cancer therapeutic development. With MCL1-specific inhibitors currently in clinical trials, it is imperative that we understand the roles that MCL1 plays in cells, especially when targeting the Bcl-2 homology 3 (BH3) pocket, the central region of MCL1 that mediates apoptotic regulation. Here, we establish that MCL1 has a direct role in controlling p73 transcriptional activity, which modulates target genes associated with DNA damage response, apoptosis, and cell cycle progression. This interaction is mediated through the reverse BH3 (rBH3) motif in the p73 tetramerization domain, which restricts p73 assembly on DNA. Here, we provide a novel mechanism for protein-level regulation of p73 transcriptional activity by MCL1, while also framing a foundation for studying MCL1 inhibitors in combination with platinum-based chemotherapeutics. More broadly, this work expands the role of Bcl-2 family signaling beyond cell fate regulation.

## Introduction

Complex decisions of cell fate occur in response to genomic stress, mutation, nutrient deprivation, and hypoxia. In response to extensive or irreparable stress, cells maintain the ability to commit suicide through a process of programmed cell death called intrinsic apoptosis. Deciding whether cellular aberrations represent a significant impedance to the integrity of the cell requires a delicate balance between cell survival and cell death. At the heart of the decision to initiate intrinsic apoptosis lies the B-cell lymphoma 2 (Bcl-2) family of pro-apoptotic and anti-apoptotic proteins^1^. Bcl-2 family members take part in a complex interaction network that regulates the integrity of the outer mitochondrial membrane. Ultimately, the Bcl-2 family is the gate-keeper for cell survival^1^.

Members of the Bcl-2 family are sub-divided into three classes based on their role in apoptotic regulation: the anti-apoptotic proteins, the pore-forming effector proteins, and the pro-apoptotic Bcl-2 homology 3 (BH3) only proteins. Despite these three classes, all Bcl-2 family members share a core amphipathic alpha-helical BH3-motif that mediates binding between family members of opposing roles^2^. The anti-apoptotic members (BCL2, BCLxL, MCL1, BCLW, and BFL1/A1) have a conserved tertiary fold that forms a hydrophobic binding groove. This groove mediates interactions with the BH3 motif that is accessible in the pro-apoptotic members^3^. When the anti-apoptotic members bind and sequester the pro-apoptotic proteins through this canonical BH3-mediated protein–protein interaction, the cell is void of apoptotic signaling. In response to intrinsic stress, the cell transiently upregulates expression or stabilization of the pro-apoptotic Bcl-2 proteins, while concomitantly downregulating the expression of anti-apoptotic members^4^. When pro-apoptotic expression surpasses that of the anti-apoptotic proteins, this allows the pore-forming effectors, BAK and BAX, to be released and induces their oligomerization. Effector oligomerization creates pores in the outer mitochondrial membrane (i.e. MOMP), which leads to release of cytochrome c into the cytosol, caspase activation, and apoptotic body formation^1,2,4^.

Over the past 15 years, targeting the anti-apoptotic Bcl-2 family members with small molecule BH3 mimetics has emerged as an anti-cancer therapeutic strategy to sensitize cells to intrinsic stress^5–7^. However, small molecules which were initially designed to target BCLW, BCLxL, and/or BCL2 have shown an acquired resistance due to the upregulation of MCL1^8,9^. In addition, more recent studies have directly highlighted MCL1 amplification as a common driver of tumorigenesis^10^. We therefore searched for novel binding sequences to strategically inhibit MCL1 using phage display. Through this screen, we identified two novel sequences that specifically bind MCL1 over the other anti-apoptotic Bcl-2 family members. These sequences are a reversal of the homologous organization of hydrophobic and acidic amino acid residues within the canonical BH3 sequence (Table 1)^11^. Therefore, we refer to these sequences as having a reverse-BH3 or rBH3 motif. Using BLAST sequence analysis of the human proteome, we identified a series of native proteins that contain rBH3-like sequences, thereby establishing a pool of potential novel MCL1-binding partners^11,12^.

**Table 1:**
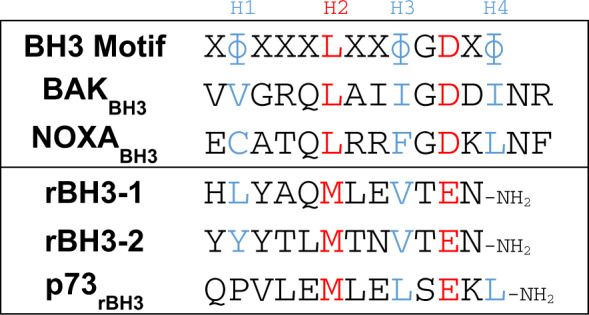
Sequence comparison between the canonical BH3 motif and the putative reverse BH3 (rBH3) motif.

One of the rBH3-containing proteins identified is p73, a transcription factor in the p53 family of tumor suppressors^11,13^. Like p53, p73 plays a role in DNA damage response, cell cycle progression, and apoptosis through transcriptional activation of target gene expression^14,15^. In p53-mutant cells, which is the case for >50% of human cancers, p73 has been suggested to play a critical role in executing these tumor suppressive functions^14,16,17^. Structurally, p73 has four core domains: a transactivation domain (TAD), a DNA-binding domain (DBD), a tetramerization domain (TD), and a sterile alpha motif (SAM)^15^. While there is sequence conservation between p53 and p73 among their TAD, DBD, and TD domains, p73 has a higher level of complexity due to its alternative promoter usage and C-terminal alternative splicing^17^. Importantly, p73 has two promoters that generate functionally distinct N-terminal isoforms from a single gene^18^. The full-length isoform from the P1 promotor contains a complete TAD (TAp73) and functions similarly to p53, while transcription initiating at the P2 promoter, located in intron 3, gives rise to a truncated protein with 13 unique amino acids on its N-terminus (ΔNp73)^19^. ΔNp73 acts as a dominant negative regulator over TAp73, thus the two N-terminal p73 isoforms perform opposing roles in the cell^20,21^. Furthermore, both the full length TAp73 and the truncated ΔNp73 isoforms can be alternatively spliced at their C-termini (ΔN/TAp73*α*, *β*, *δ*, *ε*, *ϕ*, *γ*, *η*), generating up to seven C-terminal isoforms per N-terminal variant. There are two additional alternatively spliced N-terminal isoforms, ΔEx2-p73 and ΔEx2/3-p73, generating up to 28 hypothetical p73 variants in total^19^, many of which are currently of unknown significance^22^. All of these p73 isoforms retain their DBD and the TD, suggesting that they all maintain the ability to bind DNA and associate with one another^19,22^.

While MCL1 and p73 are both known regulators of cell fate, these two proteins have never been shown to bind through a direct protein–protein interaction. Here, we confirm the direct interaction between the rBH3-containing TD of p73 and the hydrophobic BH3-binding pocket of MCL1. Further, we demonstrate that the interaction between MCL1 and p73 inhibits p73 binding to DNA, thereby acting as a novel p73 transcriptional suppressor. This work develops a unique mechanism for the cross-communication between the Bcl-2 family-mediated regulation of intrinsic apoptosis and the p73 tumor suppressive transcriptional program.

## Results

### p73 contains a putative rBH3-motif in the TD

Based on our previously published phage display screen that identified the putative reverse BH3 (rBH3) motif and its specific association with MCL1^11^, we sought to determine if rBH3 sequences found in native proteins are able to mediate direct interactions with the Bcl-2 family. BLAST analysis of the human proteome for rBH3-1 homologous sequences identified a putative rBH3 motif in the TD of p73^11^ (Table 1). Consistent with the rBH3-1 and rBH3-2 sequences identified in the phage display, the p73 rBH3 sequence contains three of the four conserved hydrophobic amino acids found in the homologous BH3 motif (H2, H3, and H4 listed in Table 1). These hydrophobic residues in the canonical BH3 motif mediate binding to the p2, p3, and p4 hydrophobic pockets previously described in the MCL1 BH3 groove^5,23^. This includes the placement of a conserved methionine at the hydrophobic position H2 which was observed to be integral in maintaining MCL1 affinity. In addition, the rBH3 sequence in p73 employs a homologous substitution for the conserved aspartic acid residue between H3 and H4, which is a persistent substitution observed in the other rBH3 sequences (i.e. aspartic acid in the BH3 versus glutamic acid in the rBH3)^11^ (Table 1).

### All native p73 isoforms can interact with MCL1

**Fig. 1:**
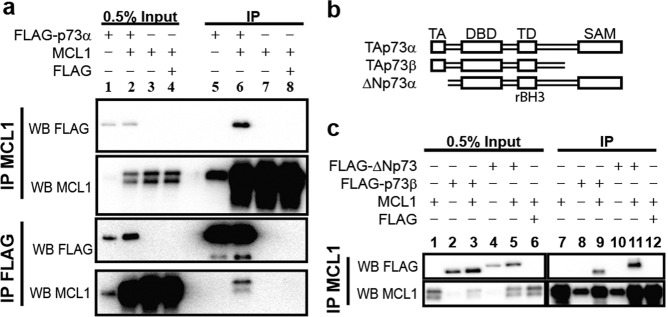
MCL1 interacts with native p73 isoforms. **a** MCL1 and either FLAG-TAp73*α* or a FLAG control were transiently overexpressed in HEK293T cells. Reciprocal co-immunoprecipatations were performed with either anti-MCL1 captured by Protein G dynabeads or with pre-conjugated anti-FLAG magnetic beads. Western blot analysis was used to confirm protein overexpression and reciprocal pulldown of full length p73 or MCL1 protein. **b** Visual representation of the domain structure of the native p73 constructs. **c** MCL1 and either FLAG-ΔNp73, FLAG-p73*β*, or a FLAG control were transiently overexpressed in HEK293T cells. MCL1 was immunoprecipitated and western blots were analyzed for either the FLAG tag or MCL1. Only lanes expressing FLAG-p73*β* and FLAG-ΔNp73 co-immunoprecipiated with MCL1.

Based on the observation that p73 contains an rBH3 sequence, we sought to determine if MCL1 and p73 interact in a cell culture model system. To assess this, we co-transfected HEK-293T cells with plasmids that express MCL1 and FLAG-tagged TAp73*α* (Fig. 1a). Following co-transfection, MCL1 and p73 proteins were co-immunoprecipated (Co-IP) in the resulting HEK-293T lysates through pulldown of either MCL1 or the FLAG-tag attached to the p73 protein. Protein immunoprecipitation (IP) was analyzed through western blot analysis. We observed that IP of either MCL1 or the FLAG-TAp73*α* resulted in pull-down of the partnered protein. As a negative control, a transfection containing the FLAG tag alone did not Co-IP MCL1 protein.

We then assessed the ability of two additional native p73 isoforms to IP with MCL1 (ΔNp73*α* and TAp73*β*) (Fig. 1b). As these isoforms lack either the TAD (ΔNp73*α*) or the SAM (TAp73*β*) (Fig. 1b)^22^, they aid in defining the p73 domain responsible for binding to MCL1. We observed that both ΔNp73*α* and TAp73*β* Co-IP with the pulldown of MCL1 protein (Fig. 1c). These studies demonstrate that MCL1 interacts with p73 in cell lysates. In addition, they demonstrate the ability for all three native p73 isoforms to bind to MCL1 thereby reducing the binding of p73 to its DBD and/or the rBH3-containing TD in HEK-293T cells. As the DBD and the TD are conserved amongst all p73 isoforms, this would suggest that MCL1 can bind to all native p73 protein variants^19^.

### The rBH3-containing TD of p73 mediates binding to MCL1

**Fig. 2:**
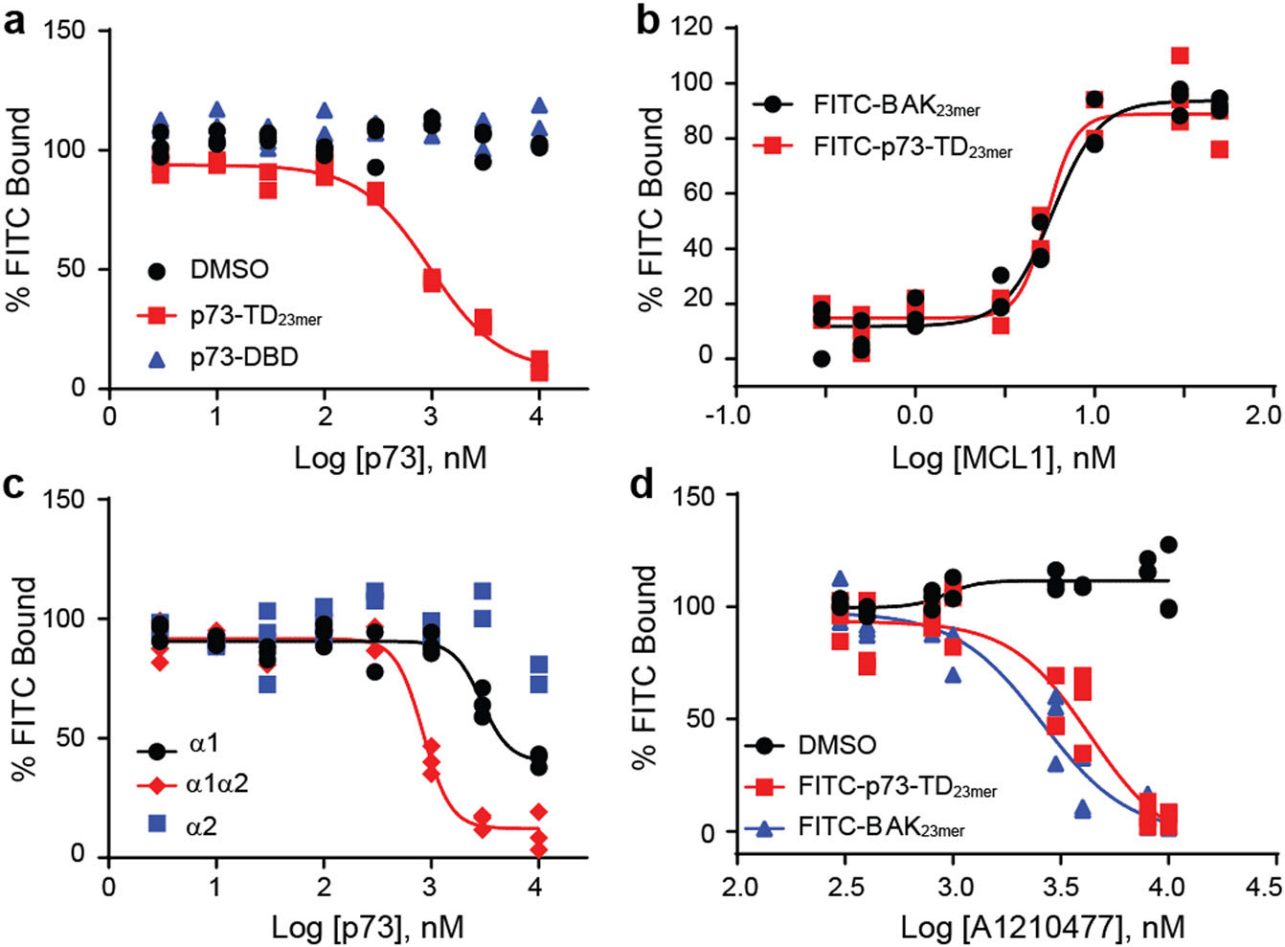
The p73_TD_*α*1 helix contains the rBH3 sequence that mediates binding to MCL1. **a** and **c** Competitive FPA consisting of 50 nM recombinant MCL1, 10 nM F-BAK, and an unlabeled titration of the p73_TD_-derived peptides. **a** The p73_23mer_ (red) is representative of the TD versus the full length p73 DBD (blue). A constant concentration of DMSO is used as a negative control (black). **b** Direct FPA with two N-terminally labeled FITC peptides, the known MCL1-binding partner BAK (black) and p73_TD_-derived peptide p73_23mer_ (red). The *K*_D_ value for both peptides is ≤5 nM, which is at or approaching the limit of detection. **c** Competitor peptides include examples of the secondary structural components *α*1 (black), *α*2 (blue), or a combinatory peptide, *α*1*α*2 (red). **d** Competitive FPA consisting of 50 nM recombinant MCL1, 10 nM F-p73_23mer_ (red, or 10 nM F-BAK (blue), and a titration of MCL1-specific inhibitor A1210477. A DMSO vehicle control is labeled in black. The plot shown is one representative assay in technical triplicate. The IC_50_s for F-p73 and F-BAK were 4.336 and 2.590 μM, respectively. All FPAs were performed in biological and technical triplicate. Each plot shows the three data points from one representative assay. All statistical analysis with the standard deviation for each peptide is listed in Table 2.

We next sought to determine which core domain, the DBD or the TD, of p73 mediates binding to MCL1. We were unable to express either of these protein domains independently in cell culture models due to protein stability and therefore moved to in vitro biochemical methods to characterize the protein–protein interaction. Prior studies have demonstrated that the DBD of p53 is able to directly interact with another anti-apoptotic protein, BCL2^24^. Therefore, we expressed and purified recombinant p73_DBD_ and tested its ability to associate with MCL1 using competitive fluorescence polarization assay (FPA) analysis and NMR chemical shift perturbation (CSP) studies (Fig. 2a, Supplementary Fig. 1). FPA is a biochemical assay that can either determine the binding constant between a recombinant protein and a fluorescently labeled small molecule or peptide (i.e. direct FPA as a measure of *K*_D_) or it can determine the concentration in which an unknown binding partner can compete with bound fluorescently labeled ligand (competitive FPA as a measure of IC_50_). Neither NMR nor competitive FPA analysis exhibited significant changes that would indicate that the p73_DBD_ associates with MCL1. Consequently, we focused our subsequent studies on the rBH3-containing p73_TD_.

To study the p73_TD_, we synthesized a corresponding 23 amino acid peptide (p73_23mer_) that parallels the homologous BH3 peptides used for other Bcl-2 family binding studies^12,25,26^ (Table 2). Through an N-terminally labeled FITC-p73_23mer_ peptide, we determined that p73 binds MCL1 with a *K*_D_ that is ≤5 nM (limit of detection for our probe) (Fig. 2b). This is comparable to the *K*_D_ of the BH3 peptide derived from BAK that has previously been used as a standard model for anti-apoptotic Bcl-2 family-binding studies^26^ (Fig. 2b). Although the exact binding constant could not be determined, a direct FPA *K*_D_ of ≤5 nM indicates that these are both high affinity-binding proteins.

**Table 2:**
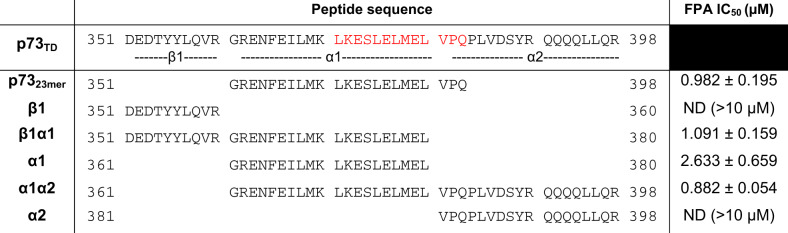
Fluorescence polarization assay results with p73_TD_-derived peptides reduce MCL1 binding to the rBH3-containing *α*1 helix of p73 (indicated in bold).

The full length p73_TD_ is composed of three secondary structural units (*β*1, *α*1, *α*2)^27^. To determine the key components of the p73_TD_ that mediates its interaction with MCL1, we prepared a series of p73_TD_-derived peptides. These peptides encompass the structural components of the single p73_TD_ monomer (*β*1, *α*1, *α*2) or combinations thereof (*β*1*α*1, *α*1*α*2)^27^ (Table 2). Using a competitive FPA, we tested each peptides’ binding to MCL1 in competition with a 23 amino acid peptide derived from the known binding partner, BAK (FITC-AHX-BAK or F-BAK)^28^. The IC_50_ of the p73_23mer_ binding to MCL1 in competition with F-BAK is 0.982 μM. Similarly, all of the *α*1, rBH3-containing peptides including *α*1, *β*1*α*1, and *α*1*α*2 exhibited comparable competition with F-BAK to the p73_23mer_ (Table 2). As demonstrated in Fig. 2c, the complete binding of the *α*1-peptide alone is not fully achieved in the absence of other structural components of the p73 TD. The *β*1 and *α*2 subunits likely facilitate helicity of the *α*1 helix, which is consistent with the FPA results obtained with the monomer subunits versus the combinatory *β*1*α*1 or *α*1*α*2 peptides. More importantly, the peptides that did not contain the putative rBH3 sequence, *β*1 and *α*2, had no detectable binding to MCL1 (IC_50_ > 10 μM). All of the IC_50_s determined through the competitive FPA for the p73 structural subunits are summarized in Table 2, along with the corresponding peptide sequences.

**Fig. 3:**
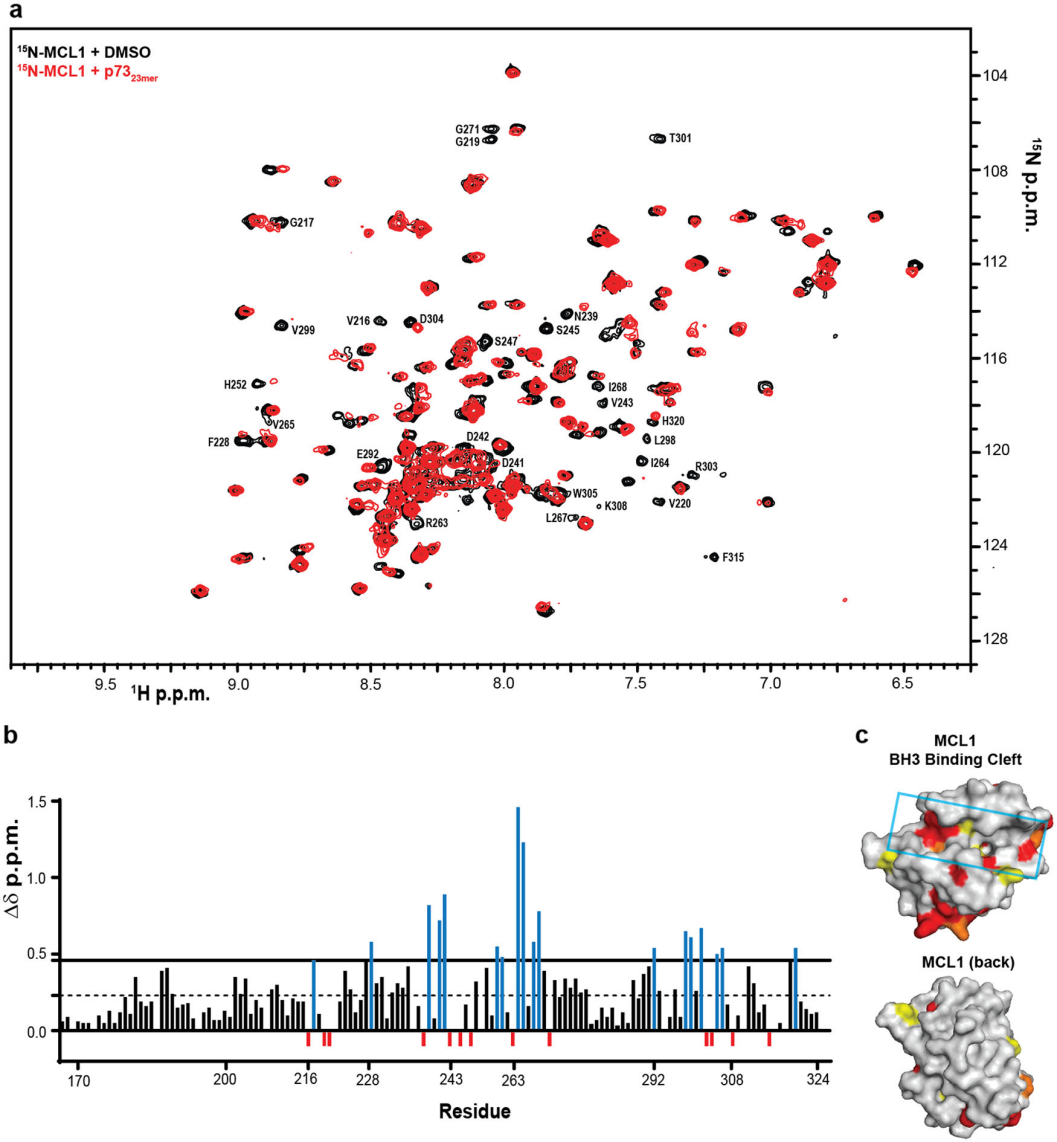
P73 binds to the BH3-binding groove of MCL1. **a** 2D [^15^N, ^1^H]-HSQC spectra of 50 μM ^15^N MCL1 + 2% DMSO (black contours) or 50 μM ^15^N MCL1 + 200 μM p73_23mer_ TD-derived peptide (red contours). Amino acids with significant CSP (Δδ > 0.046 ppm: G217, F228, N239, D241, D242, H252, I264, V265, L267, I268, E292, L298, V299, T301, D304, and H320) or residues that were unable to be identified in the bound spectra (V216, G219, V220, V243, S435, S247, R263, G271, R303, K308, and F315) are labeled for reference. **b** CSP quantified as a function of Δδ ppm for each amino acid residue. The first dotted line indicates 1SD from the mean (0.023) and the solid line indicates 2SD from the mean (0.046). Amino acids with significant CSP were identified as >2SD above the mean (blue). Amino acids that were unable to be identified in the bound spectra were set to −0.01 to differentiate them from peaks with no CSP (red). All colored amino acid peak positions are labeled in **a**. The numerical labels are used as a reference to identify significant peaks. Amino acids that were unable to be identified in the apo-MCL1 protein were not plotted as a function of Δδ ppm (residues 171, 254-261, 269, 289, 306). **c** Space filling diagram of MCL1 with the significant CSP colored as follows: Δδ ppm 0.046–0.060 (yellow), Δδ ppm 0.061–0.146 (orange), unable to be identified in the p73_23mer_ spectra (red). A cluster of significant CSP is localized in or around the BH3-binding groove, which is highlighted with the cyan box (top). A 180° rotation of MCL1 shows limited CSP impact on the reverse of the protein (bottom).

Through the p73 peptide competitive FPA analysis, we concluded that the rBH3-containing *α*1 helix mediates the protein–protein interaction between the p73_TD_ and MCL1. To determine if this interaction could be inhibited with an MCL1 inhibitor, we performed a competitive FPA between MCL1 and FITC-p73_23mer_ using the MCL1-specific inhibitor A1210477 (Fig. 3d). Using this inhibitor, we were able to inhibit the MCL1–p73 interaction with an IC_50_ of 4.37 μM. These values are comparable to the competition between MCL1 and F-BAK, which has an IC_50_ of 2.59 μM in our system (Fig. 3d). Together, these studies demonstrate that the p73_TD_ binds with a biologically relevant strength to MCL1 and is comparable to known binders like the BH3-only protein BIM and the pore-forming effector BAK^26^. Additionally, the interaction between MCL1 and p73 can be inhibited using MCL1-specific inhibitors, which are now in Phase 1 clinical trials.

### p73 binds into the canonical BH3-binding pocket of MCL1

Interactions between pro-apoptotic BH3-containing proteins and MCL1 occur through a hydrophobic BH3-binding groove formed by BH motifs 1–3 of MCL1^26,29^. As the rBH3 motif in the p73_TD_ is the reverse of the canonical BH3 sequence, we hypothesized that p73 similarly binds to MCL1’s BH3-binding pocket. To localize p73 binding to MCL1, we mapped the CSP of 2D [^1^H, ^15^N]-HSQC spectra of ^15^N-labeled MCL1 following the addition of unlabeled p73_23mer_ peptide. We observed that addition of the peptide induced significant CSP primarily localized to the BH3-binding groove of MCL1 corresponding to the three hydrophobic pockets that mediate BH3 binding (homologous rBH3 residues H2, H3, and H4 in Table 1)—p2, p3, and p4. Amongst these, amino acids required for hydrophobic interactions, including the L267 of the p2 pocket, F228 of the p3 pocket, and the V265 of the p4 pocket, were all significantly perturbed^30,31^. There were also several amino acids in which the corresponding peaks had line broadening beyond detection (red in Fig. 3b and c). Amongst these, R263 is the most significant as it mediates a critical stabilizing salt bridge with the conserved acidic amino acid within the BH3 or rBH3 helices^31^ (Table 1). Additionally, V243 of the p2 pocket and V216 and V220 of the p4 pocket could no longer be detected^31^. Of note, R263 and the hydrophobic pockets p2 and p3, are core interaction sites for the emerging MCL1-specific BH3 mimetics^30,32^.

In addition to perturbations in the BH3 pocket, we also observed a cluster of residues that were impacted at the base of MCL1. This region corresponds with a loop that follows alpha-helix (*α*5) that lies in the core of the MCL1 fold and is oriented perpendicular to the BH3-binding groove. This helix has a series of residues that help form the back of the BH3-binding groove. We and others have observed that when peptides and small molecules bind to the BH3 pocket, residues on this helix are impacted and that often the perturbation of this helix extends beyond the residues that make direct contact with the ligand^33^. Thus binding to the BH3 groove also induces allosteric perturbation to the MCL1 structure through this central alpha helix.

Taken together, the biochemical data provided thus far demonstrate that MCL1 and p73 interact through a direct protein–protein interaction. This novel interaction is driven by the rBH3 motif located in the *α*1 helix of the p73_TD_. The p73_TD_ binds to the conserved BH3-binding groove of MCL1 which mediates interactions between MCL1 and the pro-apoptotic BH3-containing Bcl-2 family members^1,26,29^. Additionally, small molecule inhibitors that target MCL1 mimic the BH3-mediated interactions and would therefore target rBH3-mediated interactions such as the identified interaction with p73 herein described^34,35^.

### MCL1 and endogenous p73 exclusively co-localize in the nucleus

**Fig. 4:**
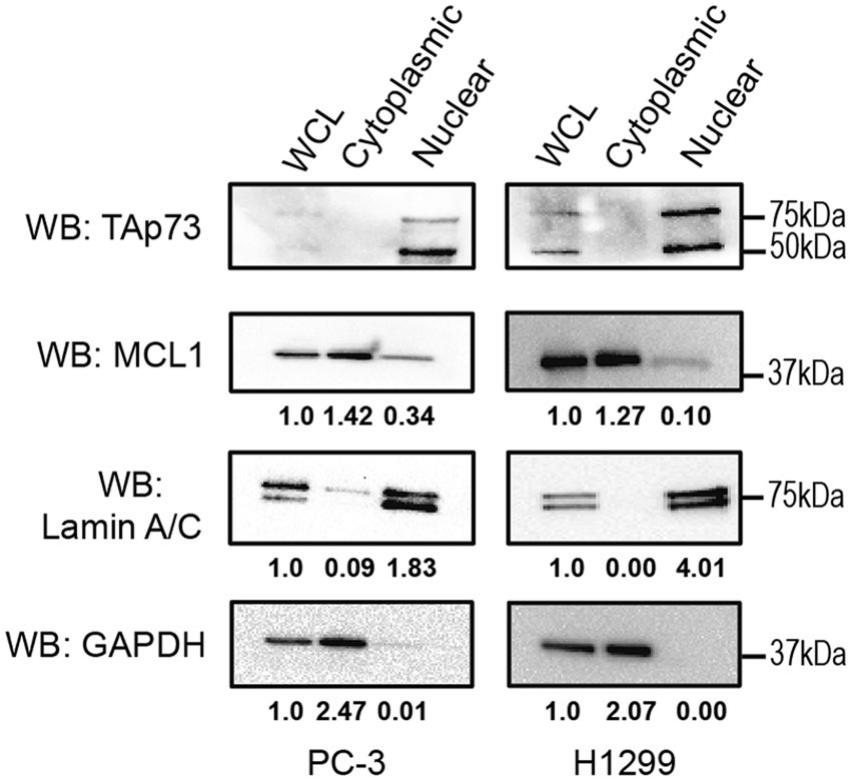
Endogenous MCL1 and full length p73 exclusively co-localize in the nucleus. Cellular fractionation of untreated PC-3 and H1299 cells into cytoplasmic and nuclear fractions. Western blot analysis was performed to determine the endogenous protein localization. GAPDH was used for a cytoplasmic control and Lamin A/C was used for a nuclear control for fractionation. The quantification provided is normalized to the whole cell lysate (WCL). Experiments were completed in biological triplicate with representative western blots shown.

Once we confirmed the direct interaction between MCL1 and p73 in cells and through various biochemical assays, we sought to determine the biological implication of this novel interaction. Canonically, the two proteins are localized in different cellular compartments with MCL1’s primary function as an anti-apoptotic member of the Bcl-2 family, positioning it outside the nucleus in the cytosol and often anchored into the outer mitochondrial membrane^1^. Yet, prior studies have observed nuclear MCL1 and proposed that it functions to regulate cell cycle progression and mediate DNA damage response through interactions with a number of nuclear proteins including IEX-1^36^ and PCNA^37^. Unlike p73, these nuclear binding partners do not contain putative rBH3 motifs and the binding sites exist in regions outside the canonical BH3-binding pocket. Likewise, while p73’s function as a transcription factor positions it predominantly in the nucleus, there have been a few studies identifying cytosolic functions of p73 and p73 fragments^38,39^. We therefore started by fractionating two cell lines into cytosolic and nuclear fractions to identify the cellular localization of our two proteins of interest in our model systems. For the remaining in vitro cell studies, we chose the p53^−/−^ cell lines, PC-3 and H1299, to separate p73 activity from the overlapping functions with its homolog, p53^40–42^. Through the fractionation studies, MCL1 was identified primarily in the cytosolic fraction, but nuclear MCL1 accounted for 26% and 13% of the total MCL1 protein population in PC-3 and H1299 cells, respectively (Fig. 4). We observed no evidence of cytosolic full-length p73 at endogenous levels or following low dose cisplatin treatment, which we utilize as a chemical agent to upregulate the p73 transcriptional activation in subsequent experiments (Fig. 4, Supplementary Fig. 2). Interestingly, we observed that treatment with cisplatin did increase MCL1 levels in both the cytoplasmic and nuclear compartments. These studies strongly suggest that the interaction between MCL1 and p73 occurs in the nucleus.

### MCL1 negatively impacts p73 DNA binding

**Fig. 5:**
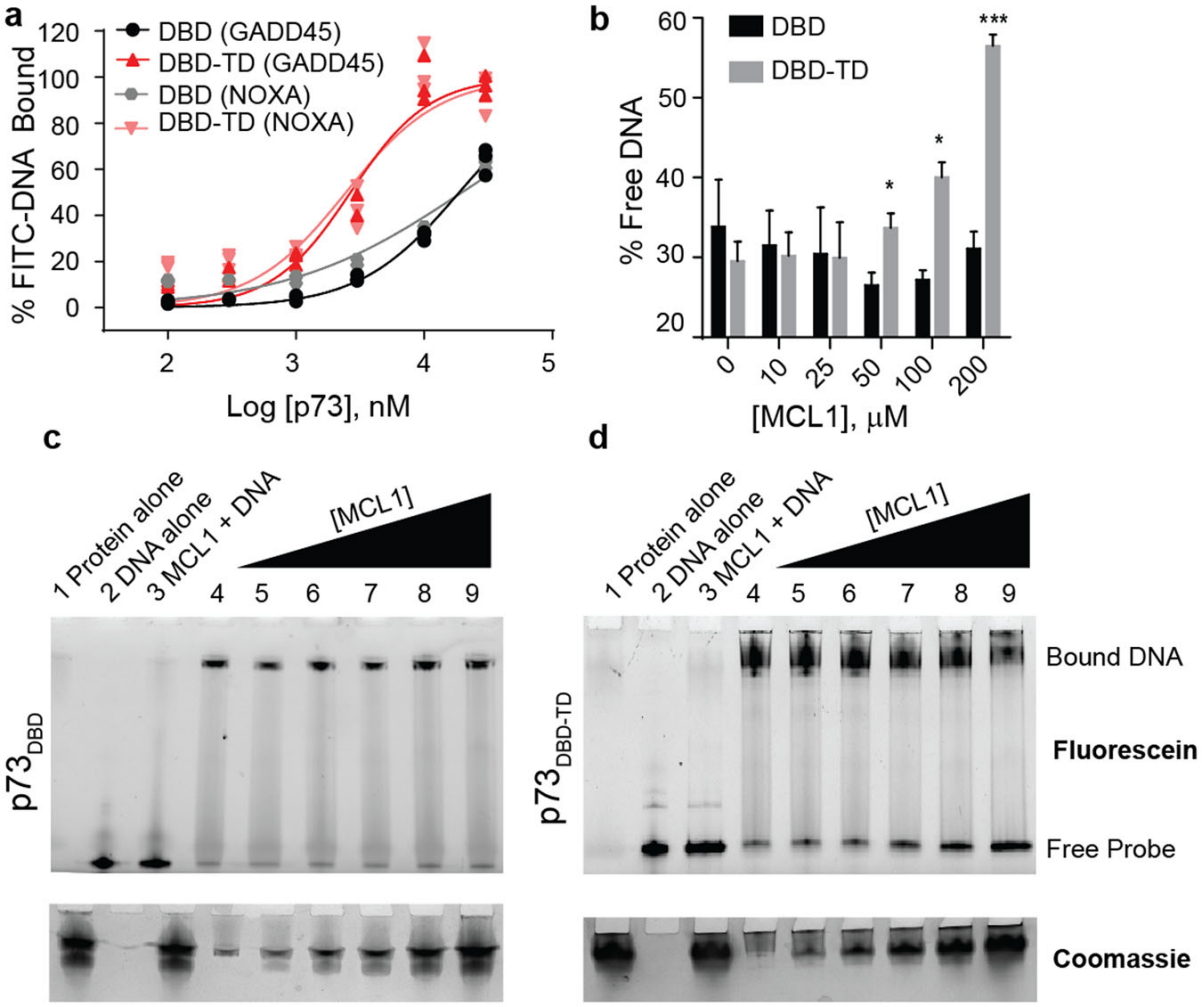
MCL1 negatively impacts p73 DNA binding in a p73_TD_-dependent manner. **a** Direct FPA between p73 (either p73_DBD_ alone or p73_DBD-TD_) and FITC-labeled DNA response elements (i.e. FITC-GADD45 or FITC-NOXA). All FPAs were performed in biological and technical triplicate. Each plot shows the three data points from one representative assay. **b** Quantification of the EMSA using p73_DBD_ or p73_DBD-TD_ with an increasing concentration of recombinant MCL1. The amount of unbound DNA was quantified through intensity values calculated by the free probe. An unpaired *t*-test was used for each concentration of MCL1 (DBD vs. DBD-TD), *N* = 3. Error is the SEM. *p*-value for 50 μM = 0.043, 100 μM = 0.0153, 200 μM = 0.0005. **c** and **d** Representative EMSAs on native TGX gels visualizing FITC-GADD45 DNA (F-DNA). **c** is the p73_DBD_ alone whereas **d** is the p73_DBD-TD_. Coomassie staining is used to visualize the protein with increasing amounts of MCL1 added into the reaction. Lanes for both gels are as follows: (1) 200 μM MCL1 alone (No F-DNA), (2) F-DNA alone (no protein), (3) 200 μM MCL1 + F-DNA, (4) 10 μM p73 protein with 1 μM F-DNA, (5)–(9) 10 μM p73 with F-DNA+ increasing concentration of MCL1 (10–200 μM).

Prior studies have suggested that the p73_TD_ increases the association of p73 with target DNA sequences^43^. To activate target genes, p73 forms a tetramer on specific DNA response elements^44^. The location of the rBH3 is at the center of the tetrameric assembly within the TD^27^. This suggests that MCL1 binding should inhibit p73 tetramer formation and sequester p73 in a monomeric or dimeric state. It should be noted that p73 dimerization can be induced through an interaction within the DBD and therefore, we do not anticipate that this interaction would inhibit all oligomeric states of p73^45^. Further, we observe that MCL1 and p73 exclusively co-localize in the nucleus (Fig. 4), thus we hypothesized that MCL1 binding to p73 should decrease affinity to target DNA sequences. Prior studies have implied that tetramer formation of p73 should increase the affinity for DNA^43^. We validated this phenomenon using recombinant p73 proteins in FPA-binding assays with two canonical FITC-labeled response elements, FITC-NOXA (F-NOXA) and FITC-GADD45 (F-GADD45)^46,47^ (Fig. 5a).

To characterize the impact that MCL1 has on p73 DNA binding due to its interaction with the p73_TD_, we utilized an in vitro DNA electrophoretic mobility shift assay (EMSA). For these studies, we used a FITC-labeled consensus response element from GADD45 (F-GADD45)^46^. To visualize the p73 DNA binding, we purified two recombinant p73 protein constructs: one that contains the DBD alone (p73_DBD_, amino acids 112–311) and another which contains both the DBD and TD that includes the rBH3 motif (p73_DBD-TD_, amino acids 112–398). In Fig. 5c, we observed that MCL1 does not impact the migration of either the free F-GADD45 DNA (lane 3) or binding of F-GADD45 DNA by p73_DBD_ (lanes 5–9). Conversely, the titration of recombinant MCL1 into a solution containing p73_DBD-TD_ and F-GADD45 DNA reduces p73 DNA binding by 27% (Fig. 5b and d). This data demonstrates that MCL1 binding to p73 can negatively impact p73 binding to canonical DNA response elements in a p73_TD_-dependent manner. It also suggests that the binding of MCL1 to the p73_TD_ inhibits the higher order oligomeric states that enhance high affinity DNA binding resulting in activation of target genes.

### MCL1 is a novel p73 transcriptional suppressor

**Fig. 6:**
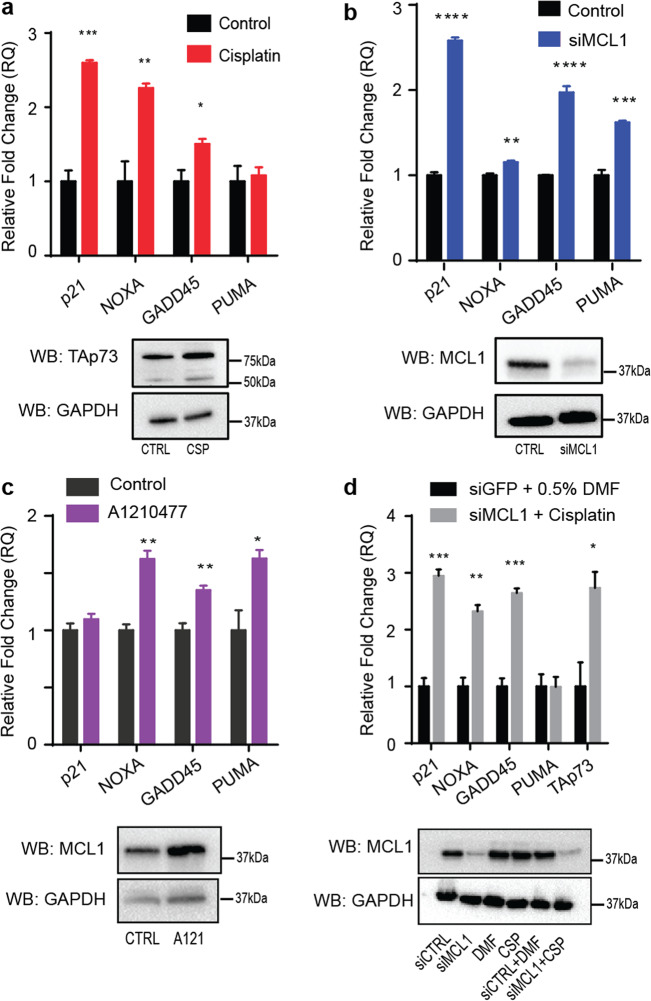
MCL1 inhibits the transcriptional function of TAp73. **a** H1299 cells were treated with 10 μM cisplatin for 24 h to upregulate endogenous p73 protein expression to generate a baseline to p73 target gene activation. Four known p73 target genes were analyzed by TaqMan RT-qPCR. After a baseline was established, H1299 cells were treated with **b** an siRNA targeting MCL1 (48 h) or **c** 2 μM MCL1 inhibitor A1210477 (24 h). The four known p73 target genes show comparable increases in comparison with the sub-lethal cisplatin. **d** H1299 cells were treated with a combination of an siRNA-targeting MCL1 (48 h) and 10 μM cisplatin (24 h). All experiments were performed in biological and technical triplicate. Bar graphs shown with SEM of one representative assay. A Student’s *t*-test was applied to each target gene for each treatment for statistical analysis, *p*-values: *p < 0.05, **p < 0.01, ***p < 0.001, ****p < 0.0001.

To determine if the MCL1–p73 interaction has an impact on the transcriptional activation of p73 target genes, we used TaqMan RT-qPCR to look at the cellular response of four known p73 target genes—p21, NOXA, GADD45, and PUMA^48–50^. For these studies, we utilized the p53^−/−^ cell lines PC-3 and H1299 to study gene expression following modulation of p73 and MCL1 protein expression. Prior studies have identified that p73 protein and/or target genes are upregulated in response to cisplatin treatment. We found that we could mimic this upregulation of p73 protein levels and/or resulting upregulation of p73 target gene activation using a sub-lethal dose of cisplatin in each cell line for 24 h. We confirmed that this dose of cisplatin did not induce apoptosis during this time frame using Annexin V/PI flow cytometry where we observed no change in the Annexin V/PI+/− cell population that is associated with apoptotic cells (Supplementary Fig. 3) Using this sub-lethal cisplatin treatment, we were able to generate a baseline for changes in the p73 target gene expression relative to an increase in activation of the native isoforms of p73 protein^51^ (Fig. 6). In both cell lines, we observed statistically significant increases in expression in at least three of the four p73 target genes (Fig. 6a, Supplementary Fig. 4). It should be noted that while both cell lines are p53^−/−^, the ratio and dependence of TAp73*α* versus TAp73*β* varies between the two cell lines. For example, as H1299 cells are predominantly driven by TAp73*α* expression and the PUMA target gene is more potently activated through TAp73*β*, it is unsurprising that PUMA is not significantly upregulated following sub-lethal cisplatin treatment in H1299 cells^52^ (Fig. 6a).

To examine if MCL1 acts as a novel p73 suppressor, we inhibited MCL1 expression using siRNA knockdown or direct protein inhibition using the MCL1-specific BH3 mimetic A1210477 (Fig. 6b and c, Supplementary Fig. 4)^53^. While BH3-mimetics inhibit the interacting binding site of MCL1, A1210477, and other MCL1 inhibitors also increase the MCL1 protein expression as they inhibit protein-binding partners that regulate MCL1 turnover^53^. Based on our hypothesis following the in vitro biochemical experiments, we anticipated an increase in p73 target gene expression would occur following inhibition of MCL1, as the knockdown would promote enhanced p73 tetramer formation and DNA binding. In each of the treatments above, we observed that when MCL1 is inhibited through a specific BH3-mimetic or knocked down, the treatment induced significant increases in gene expression in at least three of the four p73 target genes in the cell lines H1299 and PC-3 (Fig. 6b and c, Supplementary Fig. 4). The observed changes in expression were comparable to those observed following p73 activation through cellular treatment with sub-lethal cisplatin (Fig. 6a).

To verify that these findings were not an artifact of subsequent apoptotic induction following treatment with cisplatin or knockdown of MCL1, we performed Annexin V/PI staining with FACS analysis. None of the cell treatments induced a significant amount of cellular apoptosis, suggesting that the MCL1–p73 interaction is primarily responsible for these findings (Supplementary Fig. 3). This data supports our hypothesis that MCL1 can inhibit p73 transcriptional activation of target genes through a novel rBH3-mediated interaction.

## Discussion

In this study, we have described and characterized a novel protein–protein interaction between anti-apoptotic MCL1 and the p53-homolog, p73. Biochemically, we have reduced this binding to the specific elements that mediate this interaction: the rBH3 motif in the TD of p73 (p73_TD_) and the canonical hydrophobic-binding groove of MCL1. This data establishes a novel role for MCL1 where it acts as a transcriptional suppressor capable of inhibiting p73. Prior studies have highlighted how activation of p73 is essential for the transactivation of canonical response elements that regulate DNA damage response, apoptosis, and cell cycle progression^45^. Thus, MCL1’s ability to regulate p73 transactivation expands on the canonical role of MCL1 as a pro-survival and pro-proliferative protein. These findings support and expand on the role that the rBH3 motif plays in extending MCL1 function in cells. Recently, we published a study showing that MCL1 directly modulates protein stability of the cell cycle regulator P18 through a homologous rBH3-mediated interaction^12^. Our work here provides further evidence that the rBH3 motif is capable of mediating interactions with MCL1 and implies other rBH3-containing proteins should be explored.

The identified direct interaction between MCL1 and p73 poses an interesting intersection between cell survival and cell death. While this study is the first account of MCL1 modulating the activity of a transcription factor in cells, as shown in Fig. 1, we also demonstrate that MCL1 is able to bind to all alternatively spliced C-terminal isoforms as well as the dominant-negative oncogenic ΔNp73 variants. The pull-down is weaker than we may have liked, but this is not unanticipated as MCL1 has many other binding partners in the cell and p73 protein functions as a tetramer with the site of MCL1 interaction buried in this tetrameric interface. MCL1 binding may therefore impact the tetramerization of p73, an area that would be of interest for future studies as this interplay between monomeric and tetrameric p73 lies at the heart of p73 affinity to DNA, as well as the heterotetrameric regulation mediated by TA and ∆N p73 isoforms. Further, as the TD is conserved amongst all p73 isoforms, we hypothesize that MCL1 modulates all p73 activity^19^. Thus, it is possible that in cases wherein ΔNp73 variants are predominantly expressed, MCL1 would act to negatively regulate their suppressive effects and thereby activate normal p73 function. In our cell models used for these studies, we were unable to detect endogenous ΔNp73 protein using commercially available antibodies, consistent with previous accounts using these cell lines, suggesting there is no detectable endogenous ΔNp73 present^54,55^. Therefore, our studies primarily focus on the impacts that MCL1 has on the pro-apoptotic transcriptional regulation by TAp73. This alternative mechanism generates additional avenues to be explored in regard to ΔNp73 regulation.

MCL1 as a nuclear protein is not a novel finding. In 2010, a study conducted by Pawlikowska and colleagues elucidated a role for nuclear MCL1 accumulation in response to DNA damage^36^. Following genotoxic stress, early-response gene product IEX-1 is upregulated by ataxia telangiectasia mutant (ATM) and acts as a carrier protein to promote MCL1 nuclear translocation. They identified that MCL1 was an integral component of the DNA damage response pathway as the knockdown of MCL1 increased the sensitivity to genotoxic stress^39^. While we show that p73 interacts with the canonical BH3-binding pocket of MCL1, the interaction site between MCL1 and IEX-1 is localized to the C-terminal MCL1 transmembrane domain and thus, these sites are not mutually exclusive^36^. Therefore, there could be a scenario in which MCL1 has dual binding to both proteins, bound to IEX-1 at the C-terminus and to p73 through its BH3-binding pocket.

Consistent with the findings from Pawlikowska and colleagues above, several other studies over the past decade have elucidated a role for MCL1 in DNA repair, consistently reaching the conclusion that knockdown of MCL1 inhibits double stand break (DSB) repair and improves sensitivity to genotoxic therapies^56,57^. Through these studies, it has been shown that MCL1 colocalizes with DSB-induced 53BP1 foci^56^. It has also been published that ΔNp73*β* binds 53BP1^58^. Based on the data shown here, it could be proposed that MCL1 localizes to sites of DSBs through a MCL1–ΔNp73*β*–53BP1 complex. Mattoo and colleagues proposed a model in which the knockdown of MCL1 increases 53BP1 at DSBs, which inhibits homologous recombination via impaired BRCA1 recruitment^56^. To support previously suggested hypotheses and the proposed above, we suggest that the knockdown of MCL1 may free the ΔNp73*β*–53BP1 complex to bind at these DSBs. This mechanism could be exploited to target vulnerable populations with genotoxic anti-cancer therapies in combination with emerging MCL1 inhibitors^32^.

In addition to several DNA damage response studies, nuclear MCL1 has also been implicated in cell cycle regulation^12,37,59^. In 2000, Fujise and colleagues identified that MCL1 interacts with proliferating cell nuclear antigen (PCNA) in the nucleus to regulate cell cycle progression through S-phase. Like the MCL1 interaction defined here, the interaction between MCL1 and PCNA was shown to be exclusive to MCL1 supporting the idea that MCL1 has a unique role in the nucleus that is not redundant amongst other anti-apoptotic Bcl-2 family members^37^. Additionally, snMCL1 was shown to bind to CDK1 to regulate the G2/M transition^59^.

Outside its role in general cellular homeostasis, p73 expression has been evaluated in several cancer cell types and patient tumor tissues. While not commonly mutated like p53, p73 isoform dysregulation has been described in various cancer lineages in both solid and hematopoietic malignancies^60^. In the absence of wild type p53, there is a functional redundancy amongst the p53 family^13,17^. For cancers that rely on TAp73 for efficacy of chemotherapeutics such as platinum reagents, dose-limiting toxicities and resistance remain a therapeutic hurdle^61–64^. Based on the novel interaction characterized here, there is a foundation to characterize MCL1 inhibition in combination with cisplatin and/or carboplatin. By targeting MCL1 with a specific BH3 mimetic such as AZD5991 (AstraZeneca)^34^, AMG-176 (Amgen)^35^, or S64315 (Servier/Novartis)^32^, the interaction between MCL1 and p73 would be inhibited according to Fig. 2. The BH3 mimetics identified specifically target the BH3-binding pocket and would therefore inhibit not only interactions with the pro-apoptotic Bcl-2 family members, but also all rBH3-mediated interactions^12^. All three inhibitors listed above have shown promising clinical efficacy and their or related MCL1 inhibitor impact in combination therapy is worth pursuing^32^. By combining novel MCL1 inhibitors with low dose cisplatin, we could potentially combat cisplatin resistance and/or decrease the therapeutic dosing. This rationale is supported by recent accounts that knockdown of MCL1 sensitizes cancer cells to cisplatin-induced apoptosis^65,66^.

Our work shown here defines a novel protein interaction between MCL1 and p73 and supports previous studies aimed at understanding the non-canonical role of nuclear MCL1 in DNA damage response. Here, we establish a unique function of MCL1 in transcriptional regulation that has never been previously described. As this is the second account of an rBH3-mediated interaction with MCL1, our work provides further validation that the rBH3 motif is a biologically relevant and available mechanism for mediating interactions between the Bcl-2 family and other protein signaling networks^11,12^. By characterizing these rBH3-mediated interactions, we gain a better understanding of the core homeostatic mechanisms regulating MCL1. Further, knowledge of these signaling networks will better guide combination treatment of emerging BH3-mimetic-based therapies into clinic with chemotherapeutics and other genotoxic anti-cancer agents.

## Materials and methods

### Cell culture

H1299, PC-3, and HEK293T cells were maintained in humidified atmosphere with 5% CO_2_ in RPMI-1640 medium supplemented with 10% fetal bovine serum, 2.05 mM l-glutamine, 100 units/mL each of penicillin and streptomycin, and 0.25 μg/mL of Fungizone antimycotic (Life Technologies, Grand Island, NY). Cell lines were obtained from the ATCC and are regularly validated through STR profiling at the Heflin Center Genomics Core Facility at UAB. Before lysis, cells were rinsed with sterile Dulbecco’s phosphate buffered saline (1× DPBS, Corning cellgro). Cell lysates for Western Blot and IP were prepared in RIPA lysis buffer (Pierce, 89900) or in IP lysis buffer (Pierce, 87788). Lysis buffers are supplemented with 1× Halt Protease Inhibitor Cocktail with EDTA (ThermoScientific, 1861279). All experiments in this manuscript were completed in biological triplicate.

### Transfection

All transient transfections for exogenous overexpression or RNA interference were performed using Lipofectamine 3000 or Lipfectamine RNAiMax, respectively (Invitrogen). All expression constructs for exogenous overexpression were made in pcDNA3.1 plasmid. Full length human MCL1 (clone ID 3138465) and p73*α* (clone ID 40125802) cDNA clones were obtained from Invitrogen. Coding sequences were amplified with Failsafe polymerase (Epicentre, Madison, WI) and tagged with N-terminal HA and FLAG tag, respectively, using primers: forward AGAATGGGATACCCATACGATGTTCCAGAT TACGCTTTTGGCCTCAAAAGAAACG and reverse CTATCTTATTAGATATGCCAAACCAGC for MCL1 and forward AGAATGGGAGATTACAAGGATGACGATGACAAGGCCCAGTCCACCGCCACC and reverse TCAGTGGATCTCGGCCTCC for FLAG-p73*α*. The fragments were inserted into the plasmid using TOPO cloning method (Invitrogen). ΔNp73 and TAp73*β* constructs were made by deletion using Phusion site-directed mutagenesis kit (Thermo Scientific) using as a template pcDNA3.1 containing FLAG-p73*α* insert. TAp73*β* was amplified with the following primers: reverse TCAGGGCCCCCAGGTCCTGACGAGGCTGGGGTCGGCGTGGTAG, which includes fragment coding for C-terminal amino acid sequence ArgThrTrpGlyPro that is distinct from the p73*α* isoform and forward primer complementary to vector sequence, TGAAGGGCAATTCTGCAGATATCC. To make ΔNp73*α* construct forward primer CTGTACGTCGGTGACCCCGCACGGCACC TCGCCACGGCCCAGTTCAATCTGCTGAGCAGCAC and reverse primer CTTGTCATCGTCAT CCTTGTAATCTCCCATTCTaag were used. The correct sequence was confirmed for all constructs by Sanger sequencing. All RNA interference experiments were performed by the manufacturer’s protocol for Lipfectamine RNAiMAX. The final concentration of siRNA was 10 pmol/well for a 12-well dish. Silencer Select siRNA (Ambion) sequences for MCL1 are as follows: siMCL1 #1 (s8585)—GTAATTAGGAACCTGTTTCtt and siMCL1 #2 (s8583)—CCAGUAUACUUCUUAGAAAtt.

### Immunoprecipitation

Four micrograms of pcDNA3.1 plasmid DNA were premixed with 500 μL OptiMEM, 16 μL reagent P3000, and 10 μL Lipofectamine 3000 (Invitrogen). The mixture was incubated for 10 min at RT. Transfection mix was then added to cells in fresh RPMI-1640 medium supplemented with 10% fetal bovine serum, without antibiotics. 24 h after transfection, the medium was removed and fresh medium containing 50 nM bortezomib diluted from DMSO stock was added. Cells were harvested after another 24 h incubation and approximately 500 μg total protein was used for IP. The day before transfection, HEK-293T cells were seeded at 2.5 × 10^6^ on 6 cm cell culture dish. Mouse monoclonal IgG was used for MCL1 IP (RC-13, Santa Cruz Biotechnology) at 1 μg per sample. Following a one hour incubation with respective antibodies at 4 °C, immune complexes were captured overnight on protein G magnetic beads (Dynabeads, Invitrogen). For capturing FLAG-tagged p73 constructs, 25 μL anti-FLAG magnetic beads with covalently attached mouse monoclonal G2a kappa antibody were used (Clontech). Immune complexes were washed three times with IP buffer, followed by one wash with water. Proteins were eluted from beads with Pierce pH 2.0 IP elution buffer for 15 min at RT shaker, subsequently neutralized with neutralization buffer pH 8.5 (Pierce Classic Magnetic IP/Co-IP Kit) and denatured for 5 min at 95 °C with Laemmli sample buffer containing *β*-mercaptoethanol.

### Western blot analysis

Cells were lysed with RIPA lysis buffer (Pierce, 89900) supplemented with 1× Halt Protease Inhibitor with EDTA (ThermoScientific, 1861279). Lysates were mixed with 4× Laemmli sample buffer containing *β*-mercaptoethanol and denatured at 95 °C for 10 min. Proteins were resolved using SDS polyacrylamide gel electrophoresis at 150 V for 45 min and transferred to an activated PVDF membrane in a BioRad wet transfer system for 1 h at 100 V. Membranes were blocked in 5% w/v nonfat milk in PBS with 0.01% Tween (PBST) for 1 h. Membranes were then incubated with primary antibodies overnight at 4 °C. All antibodies were diluted into 1% milk–PBST solution. Secondary antibody was applied for 1 h at RT. Western Blots were developed using ECL2 reagent and Western Blotting Substrate (Pierce) and western blots were visualized on a BioRad ChemiDoc MP imaging system.

### Primary antibodies

*MCL1*: MCL1 protein in Fig. 1 was detected with anti-MCL1 polyclonal rabbit antibody (D35A5, Santa Cruz), diluted 1:1000. MCL1 Western Blots in subsequent figures were detected by anti-MCL1 rabbit mAb (D2W9E, Cell Signaling), diluted to 1:1000. *FLAG***:** Rat monoclonal IgG anti-Flag L5 antibodies (MA1-142, Pierce), diluted 1:10,000. *p73***:** anti-TAp73 mouse mAb (5B429, Novus) diluted 1:500. GAPDH**:** anti-GAPDH XP (R) mouse mAb (D16H11, Cell Signaling), diluted 1:1000. *Lamin A/C***:** anti-Lamin A/C rabbit polyclonal IgG (H-110, sc20681, Santa Cruz), diluted 1:1000.

### Secondary antibodies

*Rabbit***:** Goat anti-rabbit IgG-HRP, diluted 1:2000 (Cell Signaling), *Rat***:** Goat anti-rat IgG conjugated with HRP, diluted 1:100,000 (Pierce), *Mouse***:** Horse anti-mouse IgG-HRP diluted 1:2000 (Cell Signaling).

### Peptide synthesis

p73-derived peptides and the FITC-BAK peptide used in the FPA and NMR spectroscopy were synthesized using a standard, double-addition, FMOC, solid-phase peptide synthesis strategy on a Prelude peptide synthesis system (Gyros Protein Technologies, Sweden). 4-(2′,4′-dimethoxyphenyl-fmocaminmethyl)-phenoxyacetamido-methylbenzhydryl amine resin (rink amide MBHA resin, Anaspec) was swelled in *N,N*-dimethylformamide (DMF, Fisher Scientific) followed by methylene chloride (DCM, Fisher Scientific) to increase surface area availability for bonding. Using a double-addition FMOC strategy, the N-terminal FMOC on the growing peptide chain was deprotected with 0.8 M piperidine (Fisher Scientific) in DMF for 2 min and 30 s. The following amino acid (200 mM) was added to the N-terminus and activated with 0.4 M O-(1H-6-Chlorobenzotriazole-1-yl)-1,1,3,3-tetramethyluronium hexafluorophosphate (HCTU, Anaspec) in DMF for nucleophilic attack of the N-terminal peptidyl-resin. Next, 800 mM 4-methylmorpholine (NMM, Fisher Scientific) in DMF was added, and the peptidyl-resin, HCTU, NMM slurry was mixed for 30 min followed by four 30 s DMF washes. Peptidyl resin was cleaved using 88% TFA, 5% water, 5% phenol, and 2% triisopropylsilane for 180 min. The cleaved peptide was filtered by hand using the prelude reaction vessels away from the resin. Filtered, cleaved peptide was cold-ether precipitated and centrifuged at 14,000×*g* to pellet the resin-cleaved, crude peptide. Crude peptides were lyophilized and resuspended in 80% water/20% acetonitrile and purified over a Zorbax Eclipse XDB-C18 column (Agilent) on a 1260 Infinity HPLC (Agilent) with a 5–60% acetonitrile gradient. Following purification, the peptides were lyophilized and resuspended to 10 mM in DMSO. The purity of the peptide was confirmed by MALDI.

### Recombinant protein purification

MCL1 and the p73 constructs were transformed into BL21 (DE3) *E. coli* using the New England BioLabs protocol. Cells were grown in 1 L cultures (250 mL/flask) under kanamycin selection at 37 °C for 1 h. When an optical density (OD_600_) of 0.5–0.7 was obtained, the protein expression was induced with 1 M Isopropyl *β*-d-1-thiogalactopyranoside (IPTG, Fisher BioReagents). The optical density is monitored over several hours until a plateau is reached using the cuvette reading on a Nanodrop 2000c Spectrophotometer. At maximal density, the cells are harvested through centrifugation at 4700×*g*. At this point, the pellet is either frozen at −80 °C for future purification or resuspended in 20 mL protein lysis buffer. Once resuspended, the lysate is supplemented with two EDTA-free mini protease inhibitor tablets (Pierce, A32955) and lysozyme (0.25 mg/mL). For lysis, cells are subjected to probe sonication for 6–8 min on ice. Once complete, the cell debris is pelleted at 14,000×*g* and the supernatant is filtered through a 0.45 μm syringe filter (Millex). Following preparation, the protein is purified on an Biorad NGC FPLC system using nickel chromatography (1 mL HisTrap, GE Healthcare) followed by subsequent gel filtration (S100, GE Healthcare). Both column purifications were followed by a polyacrylamide gel run at 150 V for 45 min to confirm the presence of the protein of interest. Final protein identity was confirmed through MALDI. Both p73 constructs and MCL1 are in a final buffer of 1× PBS, pH 6.8.

### Direct FPA

In a flat-bottom, untreated black 96-well microplate (ThermoScientific), 90 μL recombinant protein is incubated with 10 μL 10× FITC-peptide or annealed FITC–DNA. The FITC-BAK sequence used in the peptide FPAs is FITC-Ahx-GQVGRQLAIIGDDINRRYD. The two FITC-labeled response elements were ordered from ThermoScientific and annealed in 5 mM Tris, 5 mM KCl, 0.01% Triton, 0.2 mM DTT (pH 8.6). The sequences are as follows: GADD45 (fwd): 5′-FITC-GAACATGTCTAGGCATGCTG-3′ and NOXA (fwd): 5′-FITC-GAGCGTGTCCGGGCAGGTCG-3′. The plate is covered with an opaque lid to shake for 30 min. Once complete, the plate is read using the FP-Fluorescein setting (1.0 s, CW lamp filter—F485, emission filter, F535) on a Perkin Elmer Victor X5 plate reader. The buffer used for all assays is 1× PBS, pH 7.4. All experiments were completed in technical and biological triplicate. Data plotted in Fig. 2 is one representative assay in technical triplicate.

### Competitive FPA

In a flat-bottom, untreated black 96-well microplate (ThermoScientific), 80 μL recombinant protein is incubated with 10 μL 10× unlabeled peptide or small molecule inhibitor shaking at 300 rpm for 20 min to allow for binding. Once complete, 10 μL of 10× FITC-peptide is added per well. The plate is covered with an opaque lid to shake for an additional 40 min. After 1 h incubation is complete, the plate is read using the FP-Fluorescein setting (1.0 s, CW lamp filter—F485, emission filter, F535) on a Perkin Elmer Victor X5 plate reader. The buffer used for all assays is 1× PBS, pH 7.4. All experiments were completed in technical and biological triplicate. All data plotted in Fig. 2 is one representative assay in technical triplicate.

### Nuclear magnetic resonance (NMR)

The NMR HSQC spectra were acquired using a Bruker 600 MHz magnet at the Central Alabama High Field NMR Facility. Samples were prepared the day of collection in 1× PBS, pH 6.8 supplemented with sodium azide (Fisher BioReagents) and deuterium oxide (99%, Cambridge Isotope Laboratories, Inc). For any samples containing peptides, the control spectra were also supplemented with DMSO for comparison. The resulting spectra were analyzed using Computer-Aided Resonance Assignment (CARA) and the peak lists were exported to Microsoft Excel for the CSP quantification and calculation of the mean and standard deviation. Any peaks exhibiting significant CSP (>2SD) were mapped to the space filling model of MCL1 on PyMOL, using PBD file 6QFI: Structure of human Mcl-1 in complex with BIM BH3 peptide. The BIM peptide was removed to display the BH3-binding pocket.

### Cellular localization

Cellular localization was performed using the NE-PER Nuclear and Cytoplasmic Extraction Reagent Kit (ThermoScientific, 78833) per the manufacturer’s protocol. The western blot quantification analysis was completed in the Image Lab 6.0.1 software using the Adjusted Volume tool. All assays were completed in biological triplicate with one representative image for each treatment group and/or cell line.

### Electrophoretic mobility shift assay

Recombinant p73 protein (DBD or DBD-TD) was incubated with 1 μM annealed FITC-GADD45 on ice (sequence above). After 15 min, recombinant MCL1 is added into each reaction for a total reaction volume of 10 μL for each sample. All reactions are mixed at 300 rpm in an Eppendorf MixMate plate shaker for 1 min, followed by a 15 min incubation on ice. After the second incubation, 5 μL native sample buffer (BioRad, #161-0738) was added to each reaction. Samples were run under native conditions on Any Kd Mini-PROTEAN TGX gels (BioRad, #456-9036) in cold 1× Tris/glycine running buffer (BioRad, #161-0771) for 20 min at 200 V. Gels were imaged on a BioRad ChemiDoc Imaging System for Fluorescein to visualize FITC-labeled DNA. Following imaging, gels were stained with Bio-Safe Coomassie G-250 Stain (BioRad, #1610787) per manufacturer’s protocol. Gels were imaged on the BioRad ChemiDoc Imaging System for Coomassie Blue to protein visualization. EMSAs were completed in biological triplicate with multiple protein purification preparations. The gels presented in Fig. 4 are one representative gel. The quantification and statistical analysis includes all biological triplicate values.

### RNA extraction and cDNA synthesis

RNA was purified from human cancer cell lines for RT-qPCR with the SurePrep TrueTotal RNA Purification Kit (Fisher BioReagents, BP2800-50) by the manufacturer’s protocol. The final concentration of purified RNA was measured using a Nanodrop 2000c spectrophotometer. Once RNA concentration was determined, a single step cDNA synthesis reaction was made in TempAssure PCR 8-Tube Strips (USA Scientific, 1402-2500) in technical duplicate as follows: 100 ng RNA, 16 μL nuclease-free water (Ambion, 1512103), and 4 μL qScript cDNA SuperMix (Quanta BioSciences Inc., 84034). The cDNA synthesis reaction per the qScript cDNA SuperMix protocol is 25 °C for 5 min, 42 °C for 30 min, and 85 °C for 5 min with a 4 °C hold. Concentration was determined after the cDNA synthesis on the Nanodrop 2000c spectrophotometer. cDNA was diluted to 25 ng/μL into Molecular Biology Grade Water (Corning, 46-000-CM) and stored at −20 °C for future use. Remaining RNA was stored at −80 °C.

### TaqMan RT-qPCR

All RT-qPCR reactions were performed in biological triplicate. A master mix for each target or housekeeping gene was made with 15 μL RNAse-free water, 3 μL 20× RT-qPCR Primer Mix (ThermoScientific), 12 μL 25 ng/μL cDNA, and 30 μL TaqMan Universal Master Mix II, with UNG (Applied Biosystems, 2020-02-29). The mix was aliquoted out 20 μL/well in technical triplicate with the final concentration of cDNA at 100 ng/reaction. PCR reactions were performed on an Applied Biosystems ViiA 7 Real-Time PCR System for 40 cycles starting at 25 °C. Each cycle is 50 °C for 2 min, 95 °C for 10 min, and 15 s, and 60 °C for 1 min. Temperature shifts at 1.6 °C/s. All data are collected and analyzed using Applied Biosystems QuantStudio Real-Time PCR Software. All target genes analyzed were normalized to GAPDH. All primers had FAM-MGB probes. All TaqMan primers were ordered from ThermoFisher Scientific with the target gene and Assay ID included: CDKN2A/p21 (Hs00355782_m1), PMAIP1/NOXA (Hs00560402_m1), GADD45A (Hs00169255_m1), BBC3/PUMA (Hs00248075_m1), GAPDH (Hs02758991_g1). Graphs in Fig. 6 are one representative assay in technical triplicate.

### Annexin V/PI staining with fluorescence-activated cell sorting (FACS)

Cells were seeded in a six-well plate and treated through various treatments to modulate MCL1 or p73 expression as they were in previous experiments. After the 24 or 48 h treatment, cells were collected through trypsin dissociation and washed twice with sterile DPBS. Cell number was counted on a BioRad Automated Cell Counter. After final wash, cells were pelleted at 1000×*g* for 10 min. The cell pellet was resuspended in 1× Annexin V binding buffer (BD Pharmingen, 556454) and 5 × 10^5^ cells were transferred to a FACS tube containing 5 μL propidium iodide (PI) staining solution (BD Pharmingen, 51-66211E) and 5 μL FITC-Annexin V (BD Pharmingen, 556419). Cells were incubated for 15 min in the dark. FACS was collected on a BD LSRFortessa and analyzed using FlowJo v10. Compensation controls used for analysis include unstained cells and cells stained for each individual fluorophore (FITC-Annexin V and Cy5-PI). Gating strategy eliminated cell debris and doublets through forward and side scatter plots.

### Reagents

The following reagents were used throughout the experiments above: MCL1-specific inhibitor A1210477 (SelleckChem, S7790); cisplatin (SelleckChem, S1166). All other reagents were described in the applicable section above.

### Statistical analyses

All experiments were repeated with at least three biologic replicates using two or three technical replicates, as reported, with data expressed as the mean ± SD. No samples were excluded. Differences between two data sets were calculated using a two-tailed unpaired Student *t*-test with *P* < 0.05 considered statistically significant. Statistical analysis was performed in Prism (Graphpad Inc.) or Microsoft Excel. *P < 0.05, **P < 0.01, ***P < 0.001.

## Supplementary information

Supplemental Figure Captions

Supplemental Figure 1

Supplemental Figure 2

Supplemental Figure 3

Supplemental Figure 4
