# Supplementary material for: MCL1 binds and negatively regulates the transcriptional function of tumor suppressor p73
Source: Cell Death Dis. 2020 Nov 3;11(11):946. doi: 10.1038/s41419-020-03068-7 (PMC7641127; doi:10.1038/s41419-020-03068-7)
Supplement: Supplementary file 1 — Supplemental Figure Captions [file 41419_2020_3068_MOESM1_ESM.docx]

**Supplementary Figures**

**Supplemental Fig 1. The p73 DNA binding domain does not bind to MCL1**. **a** ^15^N HSQC spectra with 50 µM ^15^N MCL1 + 2% DMSO (black) superimposed with 50 µM ^15^N MCL1 + 200 µM p73_DBD_ (blue) **b** CSP quantified as a function of δΔ p.p.m. for each amino acid residue. The two cut offs represent the 1 and 2 SD cut offs determined through the 50 µM 15N hMCL1 + 200 µM p73_23mer_ spectra in **Fig 2**. No amino acids in this spectra exhibited significant shifts designated by the 2 SD from the mean cut-off.

**Supplemental Fig 2. p73 is exclusively localized to the nuclear compartment following low dose Cisplatin treatment.** PC-3 cells were treated with 5 mM Cisplatin or the respective vehicle control, 0.5% DMF, for 24 hours. Cells were fractionated in cytosolic and nuclear compartments. Protein localization was analyzed through Western Blot analysis. Lamin A/C and GAPDH were used for fractionation controls. Band quantifications using the adjusted volume for each individual band are below for reference. All paired samples are normalized to the WCL treated with DMF alone.

**Supplemental Fig 3.** **Modulation of MCL1 or TAp73 does not induce apoptosis. a** PC-3 cells are treated with two siRNAs targeting MCL1 or an siRNA control for 48 hours. Cells were stained with Annexin V and Propidium Iodide (PI) to determine the apoptotic cell population. There is no significant increase in apoptosis with the knockdown of MCL1 over the siRNA control. **b** PC-3 cells are treated with MCL1 specific BH3 mimetic 2 μM A1210477 or the respective chemical control, 0.5% DMSO, for 24 hours. There is no increase in apoptotic induction similarly to the RNAi knockdown approach. **c** PC-3 cells are treated with 5 μM Cisplatin or the respective chemical control, 0.5% DMF, to intrinsically induce the endogenous p73 protein expression for 24 hours. As a positive control for the Annexin V/PI staining, cells treated with 50 µM Cisplatin showed a significant increase in apoptosis. All plots shown here are representative plots of the data set, N=4.

**Supplemental Fig 4. MCL1 inhibits the transcriptional function of TAp73.** **a** PC-3 cells were treated with 5 μM cisplatin to upregulate endogenous target gene activation. Four known p73 target genes were analyzed by TaqMan RT-qPCR. After a baseline was established, cells were treated with **b** an siRNA targeting MCL1 or **c** 2 μM MCL1 inhibitor A1210477. The four known p73 target genes show comparable increases following siMCL1 or A1210477 compared to cisplatin. All experiments were performed in biological and technical triplicate. Bar graphs shown with SEM of one representative assay. A Student’s t-test was applied to each target gene for each treatment for statistical analysis, p-values: * p < 0.05, ** p < 0.01, *** p < 0.001, **** p < 0.0001.
