# Supplementary figures and images for: MCL1 binds and negatively regulates the transcriptional function of tumor suppressor p73
Source: Cell Death Dis. 2020 Nov 3;11(11):946. doi: 10.1038/s41419-020-03068-7 (PMC7641127; doi:10.1038/s41419-020-03068-7)

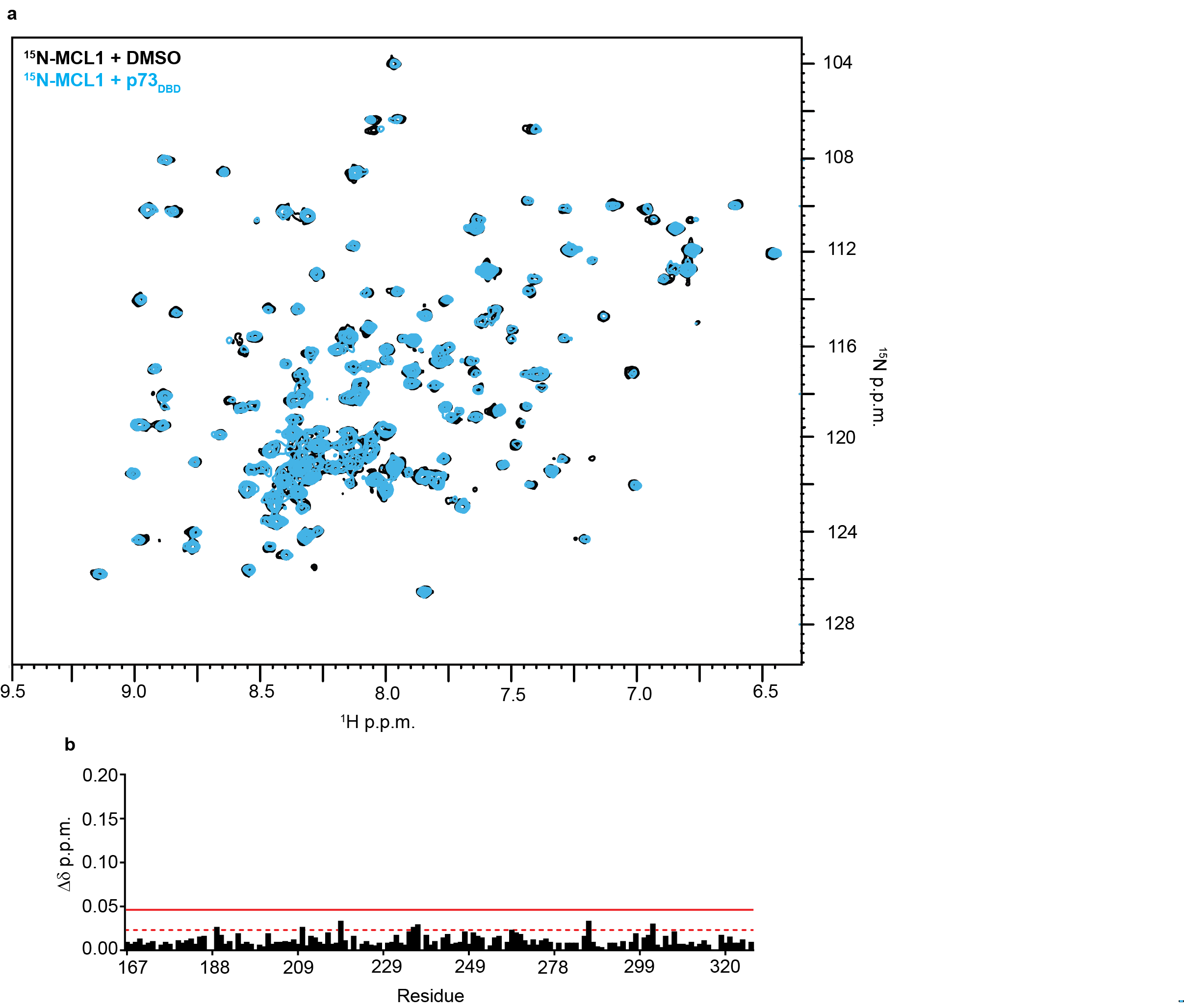

Supplement: Supplementary file 2 — Supplemental Figure 1 [file 41419_2020_3068_MOESM2_ESM.png]

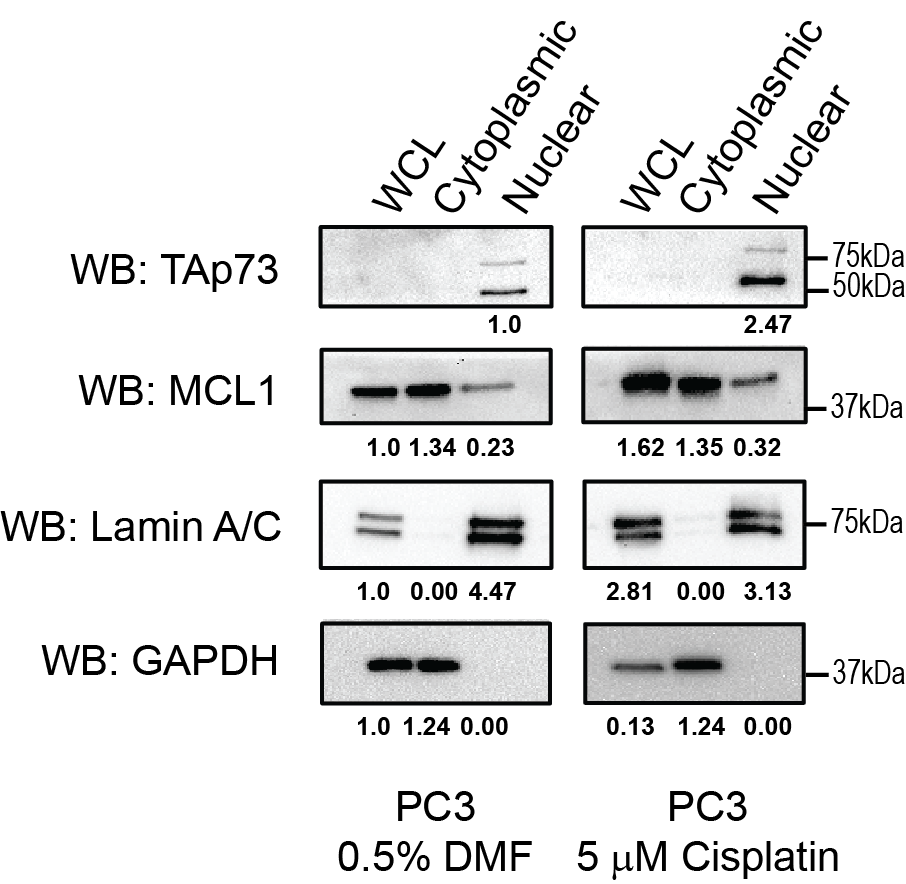

Supplement: Supplementary file 3 — Supplemental Figure 2 [file 41419_2020_3068_MOESM3_ESM.png]

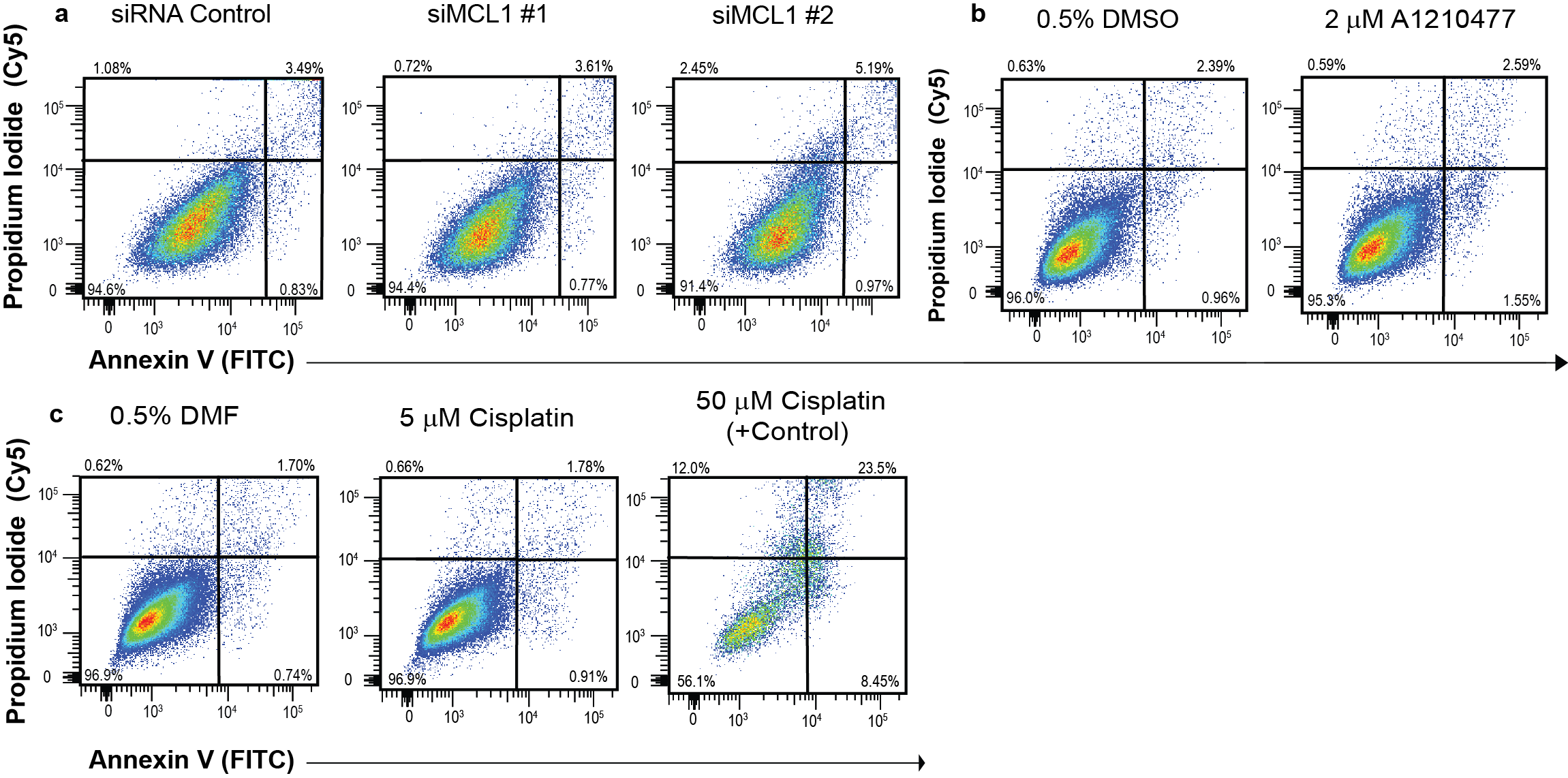

Supplement: Supplementary file 4 — Supplemental Figure 3 [file 41419_2020_3068_MOESM4_ESM.png]

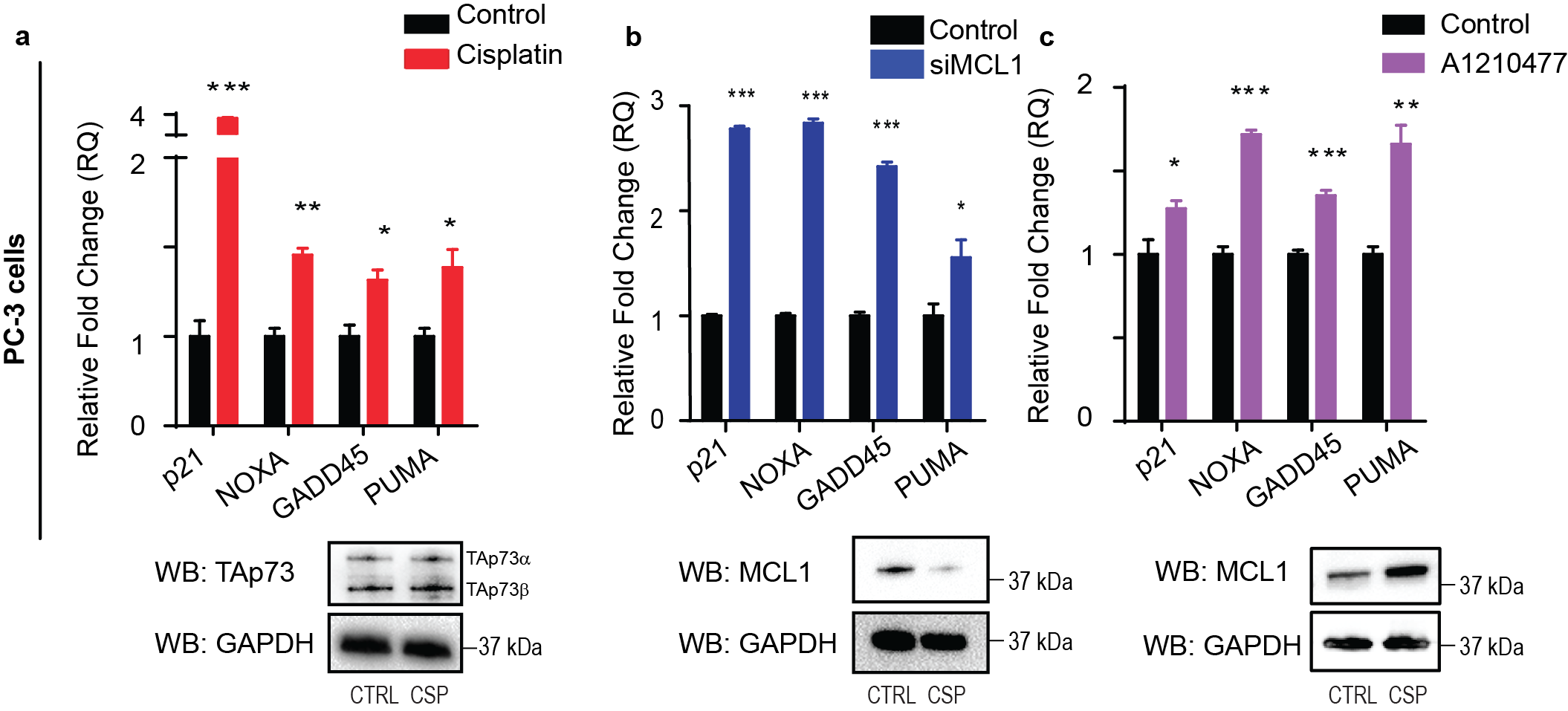

Supplement: Supplementary file 5 — Supplemental Figure 4 [file 41419_2020_3068_MOESM5_ESM.png]
